# Pig immune response to general stimulus and to porcine reproductive and respiratory syndrome virus infection: a meta-analysis approach

**DOI:** 10.1186/1471-2164-14-220

**Published:** 2013-04-03

**Authors:** Bouabid Badaoui, Christopher K Tuggle, Zhiliang Hu, James M Reecy, Tahar Ait-Ali, Anna Anselmo, Sara Botti

**Affiliations:** 1 Parco Tecnologico Padano - CERSA, Via Einstein, Lodi, 26900, Italy; 2 Department of Animal Science, and Center for Integrated Animal Genomics, Iowa State University, 2255 Kildee Hall, Ames, IA, USA; 3 The University of Edinburgh, The Roslin Institute and Royal (Dick) School of Veterinary Studies Easterbush Campus, Midlothian, EH25 9RG, UK

**Keywords:** Meta-analysis, Microarrays, Pig immune response, PRRSV infection

## Abstract

**Background:**

The availability of gene expression data that corresponds to pig immune response challenges provides compelling material for the understanding of the host immune system. Meta-analysis offers the opportunity to confirm and expand our knowledge by combining and studying at one time a vast set of independent studies creating large datasets with increased statistical power. In this study, we performed two meta-analyses of porcine transcriptomic data: i) scrutinized the global immune response to different challenges, and ii) determined the specific response to Porcine Reproductive and Respiratory Syndrome Virus (PRRSV) infection. To gain an in-depth knowledge of the pig response to PRRSV infection, we used an original approach comparing and eliminating the common genes from both meta-analyses in order to identify genes and pathways specifically involved in the PRRSV immune response. The software Pointillist was used to cope with the highly disparate data, circumventing the biases generated by the specific responses linked to single studies. Next, we used the Ingenuity Pathways Analysis (IPA) software to survey the canonical pathways, biological functions and transcription factors found to be significantly involved in the pig immune response. We used 779 chips corresponding to 29 datasets for the pig global immune response and 279 chips obtained from 6 datasets for the pig response to PRRSV infection, respectively.

**Results:**

The pig global immune response analysis showed interconnected canonical pathways involved in the regulation of translation and mitochondrial energy metabolism. Biological functions revealed in this meta-analysis were centred around translation regulation, which included protein synthesis, RNA-post transcriptional gene expression and cellular growth and proliferation. Furthermore, the oxidative phosphorylation and mitochondria dysfunctions, associated with stress signalling, were highly regulated. Transcription factors such as *MYCN*, *MYC* and *NFE2L2* were found in this analysis to be potentially involved in the regulation of the immune response.

The host specific response to PRRSV infection engendered the activation of well-defined canonical pathways in response to pathogen challenge such as TREM1, toll-like receptor and hyper-cytokinemia/ hyper-chemokinemia signalling. Furthermore, this analysis brought forth the central role of the crosstalk between innate and adaptive immune response and the regulation of anti-inflammatory response. The most significant transcription factor potentially involved in this analysis was HMGB1, which is required for the innate recognition of viral nucleic acids. Other transcription factors like interferon regulatory factors IRF1, IRF3, IRF5 and IRF8 were also involved in the pig specific response to PRRSV infection.

**Conclusions:**

This work reveals key genes, canonical pathways and biological functions involved in the pig global immune response to diverse challenges, including PRRSV infection. The powerful statistical approach led us to consolidate previous findings as well as to gain new insights into the pig immune response either to common stimuli or specifically to PRRSV infection.

## Background

Meta-analysis of microarray data combines independent microarray expression studies to create very large datasets with greater statistical power [1,2]. Compared to individual studies, a meta-analysis can provide new and stronger evidence for gene expression effects, as well as compare results from diverse studies [2]. Multiple meta-analysis approaches have been previously used and include Bayesian approach [3], GeneMeta [4], POE with Integrative Correlation [5], Fisher inverse Chi-square [6], Probability of Expression [7], mDEDS [8] andRankProd [9].

In the present study, we used Pointillist software [10,11] to perform meta-analysis of the pig immune response to diverse challenges (viruses, bacteria, non-infectious stimulus) as well as PRRSV. This software uses a modified version of Fisher inverse chi-square method for p-value combination by weighting each dataset included in the meta-analysis depending on its relevance for the system biology disturbance in question. The same software was used to study the effect of mastitis infection on ruminant immune response using a highly heterogeneous dataset [12].

Host-pathogen interactions have been the subject of intensive studies at both molecular/cellular and tissue/organism levels [13]. Specifically, Jenner and Young [14] accomplished a meta-analysis of human immune response to bacteria, viruses and immune stimulants using gene expression profiling comparisons. In the pig, data covering gene expression for immune response have accumulated and now include pig transcriptomic response to viruses, bacteria or non-infectious stimuli (Additional file 1: Table S1), which paves the way for a meta-analysis of such data. The aforementioned individual studies (Additional file 1: Table S1), as well as others, have contributed substantially to the understanding of the pig immune response [15]. Nevertheless, using meta-analysis to identify gene expression patterns shared across a myriad of experiments might contribute to a better comprehension of the biological processes related to the pig immune system.

Pigs are increasingly used as biomedical models for diseases [16]. Therefore, gaining knowledge of the porcine immune system will undoubtedly benefit many aspects of human disease research. Like other higher vertebrates, the pig immune system consists of innate and adaptive immunity [17]. Whilst innate immunity provides immediate defence against infections, adaptive immunity consists of immune responses characterized by the engagement of B and T cells in pathogen-specific protection [17,18]. Despite being less specific than the adaptive immunity, the innate immune response is critical against viruses, which keep changing their antigenic epitopes [18].

Among the major groups of innate immune effectors are interferons (IFNs) and host defences potentiators (HDPs). Whereas Type I IFNs, particularly IFN-α/β, stimulate antiviral innate immunity [19], HDPs deteriorate the viral envelope by attacking the virion glycoprotein as well as the lipid membrane [20]. Furthermore, HDPs are involved in the down-regulation of viral receptors [21] and the enhancement of adaptive immunity [22].

PRRSV is a enveloped virus with a single-stranded positive-sense RNA molecule of 14.5 kb, that harbors nine open reading frames (ORF), which encode nine viral proteins: a membrane-spanning (M) protein, nucleocapsid (N) protein, glycoproteins (GPs) and non-structural proteins (NSPs). The activation of an immune response to PRRSV occurs when porcine cells, mainly pulmonary alveolar macrophages (PAMs) and intravascular macrophages of the placenta and umbilical cord, interact with the virus [23-25]. It has been reported that PRRSV infection stimulated much less IFN-α production than did porcine coronavirus or swine influenza virus in the lungs [26], which probably leads to inadequate stimulation of antiviral immune responses and results in persistent viral infection. Furthermore, different isolates of PRRSV are dissimilar in their ability to induce IFN-α, IL-10 and IL-12 in lung or PAMs. This weakened IFN response plus increased IL-10 expression may contribute to immune modulation by some viral isolates [27,28]. Although much progress has been made in deciphering the PRRSV genetic diversity, biology and transmission routes [29-31], the results from different studies are often contradictory.

The purpose of this work was to study the pig global immune response to different challenges as well as the host response to PRRSV infection using published datasets. A new meta-analysis approach was used to assess and to confront results from diverse studies, but, most importantly, to identify particular mechanisms operating under PRRSV infection. First, all publically available datasets from immune response experiments using many different microarray platforms and pathogens (Additional file 1: Table S1), including the ones for the response to PRRSV, were considered for the first global meta-analysis. It is important to stress that the scope of this global meta-analysis was to identify commonalities among pig immune response datasets that were characterized by heterogeneity at many levels (challenge system, tissues, pathogens, etc.). Accordingly, the results of this first study determined the most common mechanisms of the pig immune response using very heterogeneous datasets and the shared mechanisms will be referred to as the pig global immune response in this report. Secondly, we performed a meta-analysis considering only the PRRS studies. Finally, we performed a functional analysis considering the genes found only in response to PRRSV and eliminating genes in common with the first global meta-analysis in order to identify particular genes and mechanisms of immunity unique to the response to PRRSV. This study elucidated possible general host-response mechanisms and specific mechanisms in response to PRRSV, confirmed previous results and highlighted new host-pathogen interaction mechanisms.

## Methods

### Gene expression datasets selection and treatment

The GEO and Array Express databases were searched for pig immune response datasets. All experiments data on pig response to diverse pathogens, time period of observation, challenge system (*in vivo*/*in vitro*), pig tissues/cells and microarray platforms (Additional file 1: Table S1), were considered for this study. We considered only the datasets of which the raw data were available except for one case whose authors provided us kindly with their unpublished raw data.

Data published before 06/07/2011, which corresponded to 809 chips from 29 separate studies, was downloaded. We used arrayQualityMetrics package of Bioconductor (http://www.bioconductor.org/) to reanalyse the quality of each dataset, which was scored on the basis of spatial, boxplot, heatmap and rle metrics. We removed any array failing in at least two metrics as performed in previous study [32]. Normalisation for each dataset was performed independently using the robust multi-array average (RMA) expression measure [33] for Affymetrix and Lowess normalization within the arrays for the other platforms (Qiagen, Operon, Custom array; see Additional file 1: Table S1).

### Meta-analysis procedures

Our main focus regarding the meta-analysis was the set of genes involved in the pig general immune response and then in the pig response to PRRSV infection. Therefore, first we used the complete dataset for pig global immune response and second only the data corresponding to PRRSV infection for the pig response to PRRSV which consisted of 6 datasets harbouring 279 chips.

To perform meta-analysis of pig microarray data, we used the software Pointillist (http://magnet.systemsbiology.net/software/Pointillist[10,11]), which is highly suitable for integrating heterogeneous datasets [10]. Pointillist infers elements affected by a perturbation of biological system after integrating and evaluating evidence that corresponds to that perturbation. The evidences in this meta-analysis are the p-values of each addressed element, which in our case represents the gene expression that corresponds to the probes (elements) on each microarray chip. Consequently, as a first step, we determined the p-value of each microarray clone using a Pointillist significance calculator approach that can analyse the probability distribution of a set of observations and compute the statistical significance of each observation on the basis of that distribution. This probability corresponds to the likelihood that a given observation could happen by chance given the global distribution for all the observations. Accordingly, we employed the cumulative density function of a nonparametric distribution with a Gaussian kernel density [34,35] to calculate the significance of the observations.

Because of the large quantity of data in the global immune response, we used the Pointillist “parametric non-weighted” method for data integration, which is suitable for the convergence. A “non-parametric weighted” method used for the PRRSV analysis was previously used [12].

During data integration, Pointillist classifies elements as “affected”, if the element’s p-value is below a chosen threshold alpha (0.05 in this study) or “non-affected” otherwise. “Combined effective significances” are calculated for each elements by weighting, normalizing, transforming, and combining the element’s specific p-values into one single element significance using a Fisher-like transformation and by finally smoothing the distribution of these significances using a smoothed Gaussian kernel density function.

For the “non-parametric weighted” method, weights used during the transforming operation are also calculated for each piece of evidence. Either in global immune or PRRSV responses, the Pointillist run contained a row for each probe having a p-value in at least 10% of the data. The false discovery rate (FDR) corrections were as set by default 0.05 and 0.10 for the “parametric non-weighted” and the “non-parametric weighted” methods, respectively. The data integration with Pointillist software takes as input the p-values calculated for each probe and outputs an integrated p-value for each probe independently of the fold change direction (up/down-regulation).

### Co-expression analysis

The goal of this analysis was to make a wise clustering of the significant genes from the two meta-analyses before performing the functional analysis. As differences in array technology and data processing create a bias in comparing transcript abundance between studies [14], we considered microarrays data from 16 studies that used the Affymetrix platform, which corresponded to 322 arrays (Additional file 1: Table S1). These arrays represents 41% (332/779) and 29% (80/279) of the complete arrays that corresponded to “Pig global immune response” and “Pig specific response to PRRSV infection”, respectively. In practice, we retrieved the fold changes expression corresponding to the significant genes in those two meta-analyses using Bioconductor “simpleaffy” package [36]. Those fold changes correspond to the gene expression ratios of specific cases relative to the control used in that specific experiment. Subsequently, we used “WGCNA” R package [37] combined with a customized R/Python to perform a co-expression clustering analysis, which consisted mainly in network construction, module detection, gene selection, calculations of topological properties and visualization.

### *Probe sequences annotation and functional analysis*

The array elements used in this analysis were matched with the Iowa Porcine Assembly (IwPA) which consist of 140087 consensus sequences (contigs), called the Iowa Tentative Consensus (ITC), and 103888 singletons [38]. Array elements that mapped to the same IwPA sequence were deemed to be comparable across microarray platforms. Subsequently, those consensus sequences were aligned to NCBI RefSeq database for orthology annotation. These mappings are available at: http://www.animalgenome.org/pig/projects/array_annotatn/. The NCBI RefSeq symbols of the affected genes were mapped to their corresponding gene names in the Ingenuity Pathways Analysis software (IPA) (“http://www.ingenuity.com/”). The corresponding lists were submitted to IPA to get the canonical pathways, biological functions and networks significantly associated with the gene lists. Ingenuity Pathways Analysis is a knowledge database and web-based analysis system that permits the creation of molecular networks, biological function and metabolic canonical pathways that are most significantly represented in genes set of interest. The p-value associated with biological process or pathway annotation is calculated according to the right-tailed Fisher Exact Test. Therefore, this statistical test assesses the null hypothesis: “is the proportion of genes that map to a particular function or pathway in my sample similar to the proportion that map in the entire population (IPA reference set)”. Only over-represented functions or pathways that are more abundant than expected by chance, are reported as significant.

We focused on the five most affected canonical pathways and biological functions that belonged to the sub-group: “Molecular and cellular functions” and networks. We used the default IPA “universe” which contain all genes and endogenous chemicals of the IPA library.

To confirm our functional analysis, we utilized an additional approach in which we performed co-expression analysis of only differentially expressed genes and these identified clusters were then analyzed separately by IPA.

## Results and discussion

### Heterogeneity of gene expression data used in pig global immune response: microarrays chip platforms, challenge systems and pig tissues/cells

We collected all publicly available microarray datasets that corresponded to pig immune response studies. The data were very heterogeneous (Additional file 1: Table S1) in term of chip platforms (e.g., Affymetrix, Qiagen, Operon, custom array), pathogens (e.g., PRRSV, Pseudorabies, Streptococcus suis), infected tissues (e.g., spleen, lung, kidney, pulmonary alveolar macrophages), and pig breeds (Landrace, Large White, Pietrain, Duroc and Wild boar). 29 microarray datasets that corresponded to 809 chips were considered in this study. Datasets quality checking identified 30 chips to be of insufficient quality for meta-analysis. Thus, for the pig global immune response, we used 779 chips that corresponded to 29 datasets; and for the pig response to PRRSV infection, we used 279 chips obtained from 6 datasets (Additional file 1: Table S1).

### Pig global immune response

To perform the meta-analysis for the pig global immune response, we used 30504 array elements across all datasets, which were aligned to custom cDNA assemblies [38] to match elements across arrays. Of the 1464 (Additional file 2: Table S2-A, p < 0.05) unique probes identified by Pointillist as being significantly altered (FDR < 0.05), 1241 (Additional file 2: Table S2-B) were mapped to NCBI gene names and 1044 were present in the IPA knowledge database (Additional file 2: Table S2-C).

**Figure 1:**
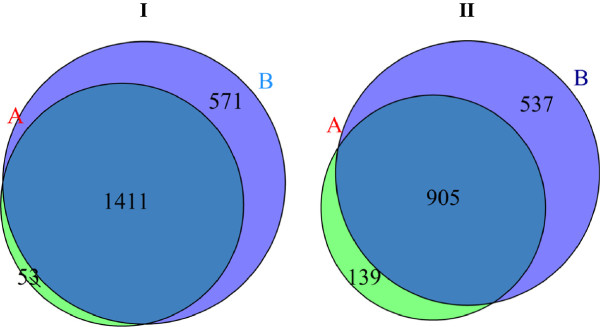
**Venn diagram illustrating the significantly affected genes in combination between the pig global immune response and pig response to PRRSV infection. I**. Venn diagram illustrating the significantly affected genes in combination between the pig global immune response (A. 1464 genes) and pig global immune response without including PRRSV datasets (B. 1988 genes). We highlighted the number of significantly affected genes in common (1411) and distinct between A and B (53 and 571 for A and B, respectively). **II.**Venn diagram illustrating the significantly affected genes in combination between the pig global immune response (A. 1044 genes) and pig response to PRRSV infection (B. 1442 genes). We highlighted the number of significantly affected genes in common (905) and distinct between A and B (139 and 537 for pig global immune response and pig response to PRRSV, respectively). The lists of corresponding genes can be found in Additional file 2: Table S2 and Additional file 5: Table S5.

From a total of 779 microarray datasets used in the pig global immune response a subset of 279 were connected to PRRSV infection. Hence, we carried out an additional global meta-analysis without the PRRSV datasets to figure out if the gene overlap between the global immune response gene set and the PRRSV gene set is due to an over-representation of PRRSV data in the global analysis. Pointillist analysis identified 1982 unique significant probes (FDR < 0.05). Interestingly, 96% (1411 out the 1464) of the probes identified previously as significant for the pig global immune response were always significant in this analysis even without the PRRS datasets (Figure 1.I). This finding shows that the output of the global immune response was not overly influenced by the PRRS datasets.

### Affected canonical pathways, biological functions and transcription factors in pig global immune response

The top five canonical pathways identified by IPA as being most significantly associated with the pig global immune response were EIF2 Signalling, Oxidative Phosphorylation, Regulation of eIF4 and p70S6K Signaling, Mitochondrial Dysfunction and mTOR Signaling (Additional file 3: Table S3A). Moreover, the five most significant biological functions identified during this meta-analysis were cell death, cellular growth and proliferation, protein synthesis, RNA-post transcriptional modification and gene expression (Additional file 4: Table S4A).

The most significant canonical pathway in our study: phosphorylation of eukaryotic initiation factor-2 (eIF2) (p = 1.03e-66) is involved in eukaryotic protein synthesis and has a central role in stress induced translation regulation [39,40]. Specifically, the phosphorylation of (eIF2) has been reported to be involved in the inhibition of cellular and viral protein synthesis [41]. Furthermore, Regulation of eIF4 and p70S6K signalling plays also critical roles in translational regulation [42]. In the gene list corresponding to this pathway, eIF4A2, eIF4A3 and PABPC1 were found together with a myriad of eukaryotic initiation factors (e.g. eIF3C, eIF2A, eIF3M), that are important intermediaries in translation initiation [43]. It is probable that the top biological functions protein synthesis, RNA-post transcriptional modification and gene expression reflect the translation regulation during the pig global immune response.

**Figure 2:**
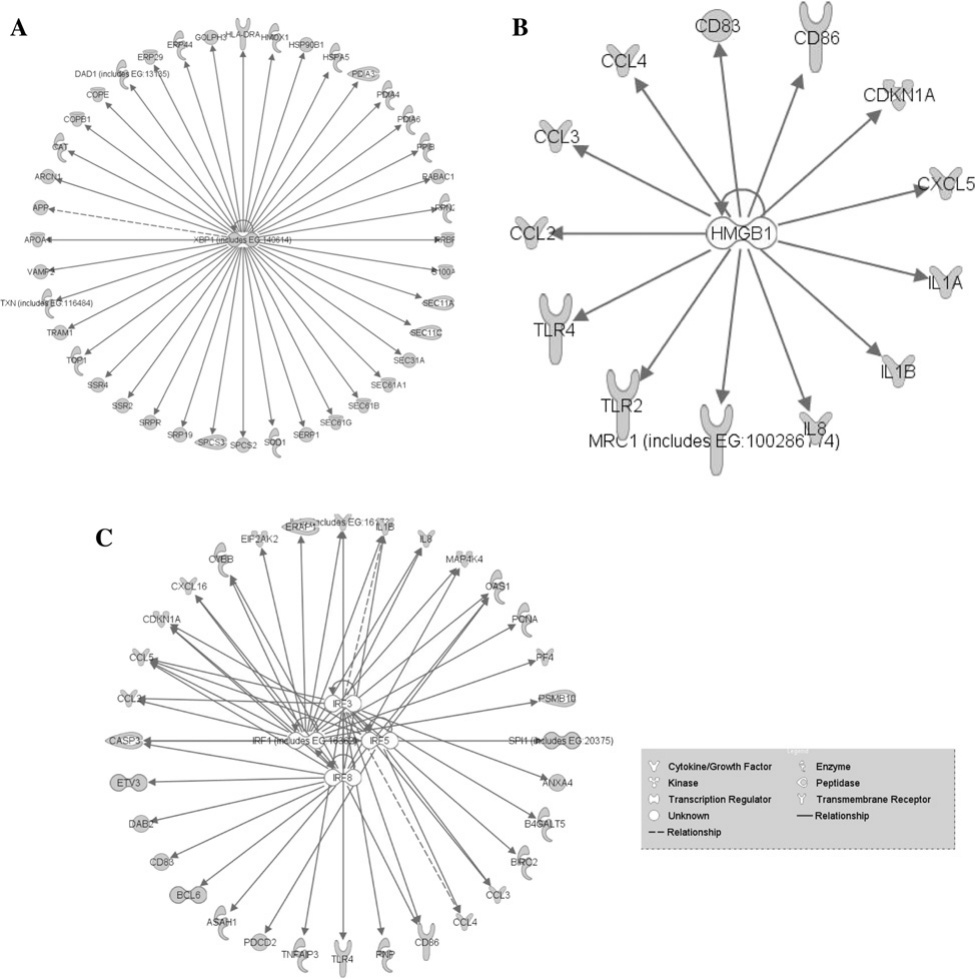
**Transcription factors and their target genes. The transcription factors estimation was done using the IPA “transcription factor estimation” feature. A**. XBP-1 transcription factor and its target genes found in the gene list corresponding to pig global immune response. Note that that XBP-1 itself was found to be differentially expressed in the gene list and had 42 target genes. **B**. HMGB1 transcription factor and its target genes found in the gene list corresponding to pig specific immune response to PRRSV infection. **C**. IRF1, IRF3, IRF5 and IRF8 transcription factors and their target genes found in the gene list corresponding to pig specific response to PRRSV.

It is well established that the alteration of the translational apparatus upon different stress situations has an upstream effector at the endoplasmic reticulum (ER) level [44]. This activates the Unfolded Protein Response (UPR) that regulates ER protein folding and plays an important role in innate immunity [45]. This response mechanism was highly corroborated in our findings. Therefore, the UPR pathway was significantly enriched (p = 0.017) in this analysis, although it was not among the top pathways and involved four genes, XBP1, ATF4, EIF2S1 and HSPA5. Moreover, the transcription factor X-box-binding protein-1 (XBP-1) that plays a central role in activating the UPR [46] was altered in this study. As well, using the transcription factor estimation of IPA, the XBP-1 transcription factor was significant (4.06E-09) and connected to forty differentially expressed gene targets (Figure 2A). In agreement with these findings, it was reported that bacteria and virus infection, as well as general stimuli, activates the UPR in host cells due to the massive production of unfolded proteins [12,45,46].

Mitochondrial dysfunction and oxidative phosphorylation, two pathways found to be activated in this analysis, are related processes. Oxidative phosphorylation leads to the phosphorylation of ADP to ATP necessary to the functioning of cellular processes, which generates reactive oxygen species (ROS) superoxide. Infections by pathogens alter cell homeostasis and cause elevation in cellular levels of ROS [47,48]. The cell death function that was the most significant molecular and cellular function (p = 1.01E-24), might be the result of oxidative stress and mitochondria dysfunction.

mTOR signalling mediates the phosphorylation of eIF4EBP1 and the ribosomal protein p70S6K, thereby affecting the translation of mRNA [49]. This observation would indicate that mTOR might be connected to the EIF2 signalling and Regulation of eIF4 and p70S6K pathways. Moreover, mTOR signalling could also be linked to mitochondria dysfunction, as it acts on mitochondrial metabolism [50]. These findings indicate that the aforementioned canonical pathways are all interconnected in the regulation of mRNA translation and mitochondrial metabolism. Taking all together, we hypothesise that the stress provoked by the stimulus affects host cell homeostasis, which prompts the ER to send signals of stress. Consistent with our results, this stress signal might occur through activation of one or more protein kinases that specifically phosphorylate the subunit α of eukaryotic translation initiation factor 2 (eIF-2a).

Previously, Jenner and Young [14] integrated a group of human gene expression datasets to study host-pathogen interaction pathways. Whilst in our analysis, the reported top canonical pathways and biological functions reflected general biological processes, most of the pathways reported in Jenner and Young were closely involved in immune responses and included among others; inflammation response, IFN-stimulated genes and TLR-mediated common and specific response [14]. These differences could be the consequence of different methods used in the two studies. While Jenner and Young used the magnitude of gene expression profiles among experiments as a key factor for clustering the genes with subsequent annotation using biological ontologies, we used a robust methodology based on an optimization algorithm that minimizes the numbers of false positives and false negatives. Furthermore, in this study we included non-infectious perturbations, which might hinder the identification of infectious specific response. Another very plausible explanation to those differences is that the general signature highlighted during the pig global immune response might be due to the sampling process. Therefore, the sampling process might induce a stress response independent of whatever previous perturbation is under investigation. In individual studies it is impossible to distinguish this sampling damage response from other responses but maybe the biggest strength of the meta analysis here is the capability to detect these inadvertent responses.

Our approach would rank genes according to their importance in the biological signal across the gene expression datasets, notwithstanding, without reporting the corresponding fold change in gene expression. Nevertheless, it is possible to retrieve the gene expression of the genes identified as significant in the meta-analysis. Accordingly, we used the fold changes from one microarray platform (Affymetrix) to make a clustering analysis using the significant genes. The co-expression analysis corresponding to the pig global immune response revealed two clusters (Additional file 5: Table S5-A, Additional file 6: Figure S1). The first and second cluster contained 884 and 359 probes, respectively. Mapping the probes within each group onto the IPA revealed that among the top five canonical pathways in cluster 1, four are among the top in the global immune response and are EIF2 Signalling, Regulation of eIF4 and p70S6K Signalling, mTOR Signalling and Mitochondrial Dysfunction. The canonical pathways expressed in the second cluster contained among others, the NRF2-mediated Oxidative Stress Response that was in the 8th position in the pig global immune response (Additional file 7: Table S6). Finding only two clusters in the set of significant genes in this meta-analysis is in concordance with the fact that the meta-analysis approach provides only commonalities among different experiments and that the more heterogeneous the data are, the less common biological pathways one can identify. Moreover, this finding reflects the strong connectivity between the significant genes in this meta-analysis.

Three transcription factors were highly significant; MYCN (p-value = 1.71E-45), MYC (p-value = 1.11E-11) and NFE2L2 (p-value = 5.97E-17) during this analysis. The visualisation of the two transcription factors (MYCN and MYC) in interactions with their target genes (Additional file 8: Figure S2) shows that these two transcription factors and their targets explain the modulation of three among the five top canonical pathways involved in pig global immune response; EIF2 Signalling, Regulation of eIF4 and p70S6K Signalling, and mTOR Signalling (highlighted in Additional file 8: Figure S2). Furthermore, NFE2L2 and its target gene group explain the alteration of the oxidative stress response highly affected during the pig global immune response (data not shown).

### Pig specific response to PRRSV infection

To study the specific immune response to PRRSV infection, we considered the significant genes responsive exclusively during PRRSV infection, eliminating the genes that were also significantly altered in the general immune response. This approach might shed light on specific and particular biological pathways to PRRSV that could be masked by more general and abundant pathways of the immune response. First, we performed meta-analysis using all the experiments when PRRSV infection occurred. The total dataset corresponded to 31353 probes in 278 chips from 6 datasets. Of the 1906 (Additional file 9: Table S7-A) probes identified by Pointillist as significantly affected by PRRSV infection, 1612 (Additional file 9: Table S7-B) were mapped to NCBI and 1442 recognised by IPA (Additional file 7: Table S7-C). To take in consideration only the genes specific to PRRSV infection, we removed from this list of 1442 IPA-recognized genes those genes present on the list of pig global immune response (905 genes) resulting in a final list of 537 genes (Figure 1.II, Additional file 10: Table S8-A).

### Affected canonical pathways, biological functions and transcription factors in pig specific response to PRRSV infection

The pig specific response to PRRSV infection was characterized by the involvement of well-defined pathways in immune response to viral infection, including TREM1 signalling, role of hyper-cytokinemia/ hyper-chemokinemia in the pathogenesis of influenza, toll-like receptor signalling, glucocorticoid receptor signalling and communication between innate and adaptive immune response (Additional file 3: Table S3B). Furthermore, the top five molecular and cellular functions in this analysis were: Cellular movement, Cell-To-cell signalling and interaction, Cellular growth and proliferation, Cell morphology and molecular transport (Additional file 4: Table S4B). TREM-1 are abundant receptors, distributed on myeloid cells including neutrophils, CD14 high monocytes/macrophages and lung alveolar macrophages [51]. Stimulation of TREM by its unknown ligand or of toll like receptor (TLR) by lipopolysacharide can provoke the association of TREM1 and TLR, activating interleukin-1 receptor-associated kinase 1 (IRAK1), NF-κB, and the pro-inflammatory response [51,52]. In this study, the modulation of TREM-1 was associated with changes in expression of chemokines (e. g. CCL2, CCL3), interleukins (IL-6, IL-18, I1-beta), and toll like receptors (TLR2, TLR4) and others molecules such as a transmembrane receptor (CD86), the molecule CD88 and the growth factor receptor bound-2 (GRB2). TREM-1 has been implicated in the amplification of septic shock by enhancing the (TLR)-mediated production of proinflammatory cytokines [53,54] in response to viruses like Marburg virus (MARV) and Ebola virus (EBOV) in humans [55]. If the involvement of TREM-1 and the role of neutrophils are confirmed during PRRSV infection by further investigations, it might be possible to improve the potency of anti-PRRSV vaccines by regulating the recruitment of myeloid cells, especially neutrophils to the site of antigen delivery.

Role of Hypercytokinemia/hyperchemokinemia in the Pathogenesis of Influenza could reflect the similarity between PRRSV and influenza viruses in the matter of hypercytokinemia and hyperchemokinemia symptoms. In this study, the activation of this pathway involved the expression of chemokines (CCL2, CCL3, CCL4, and CCL5), interleukins (IL1A, IL1B, IL8 and IL18) and the chemokine (C-C motif) receptor 1 (CCR1). This finding is in concordance with a report showing that during PRRSV infection, there is an over-production of pro-inflammatory cytokines in the lungs (IL-1, IL-6 and TNF) that is exacerbated after stimulation with LPS in PRRSV infected pigs [56].

Toll-like receptors (TLRs) are transmembrane receptors that bind to specific molecular patterns in bacteria and viruses. In our study, TLRs signalling specific to PRRSV infection was build upon 8 genes (TLR2, TLR4, LY96 ,TOLLIP, JUN, MAP3K1,TAB2, MAP4K4 and EIF2AK2). The involvement of TLRs in PRRSV infection is known; lymphoid tissues infected with PRRSV have significant alteration of TLRs, 2,3,4,7 and 8 gene expressions [57]. Moreover, TLR1, 2, 4, and 6 were significantly increased in PRRSV infected porcine lungs [58]. This finding is in concordance with our analysis in which TLR2 and TLR4 expressions were affected.

Glucocorticoids regulate a large number of immune processes by inducing transcription of anti-inflammatory genes and repression of pro-inflammatory gene transcription [59]. Many bacterial and viral infections result in an activation of the hypothalamic-pituitary-adrenal (HPA) axis and increased glucocorticoid release that could advantageous to both the infectious agent and the host [60,61].

The host defence against pathogen infection consists of a complex interplay between components of the innate and the adaptive immune system [62]. In this context, two related biological functions were exclusively affected during PRRSV infection; “cellular movement” and “cell to cell signalling and interaction”. “Cell movement” included, among others: homing, chemotaxis, attraction of myeloid cells, and cell movement of neutrophils, granulocyte, phagocyte and leukocyte, while “cell to cell signalling and interaction” included attraction of myeloid cells and granulocyte, adhesion of phagocytes, and recruitment of granulocytes and neutrophils. These biological functions might have an important role during the crosstalk between the innate and adaptive immune response.

To determine the canonical pathways under-represented in pig specific response to PRRSV infection, we used IPA to analyse the 139 genes significantly and uniquely altered in the global immune response (Additional file 10: Table S8B, Figure 1.II). Among the top biological functions of this analysis, we found cell death as the most significant, which included 56 genes, of which 40 genes were involved in apoptosis and 16 in cell survival (data not shown). This finding might suggest that the under-representation of cell death during the PRRSV infection is a strategy adopted by PRRSV to improve its infectivity. A similar result has been reported previously in which PRRSV stimulates anti-apoptotic pathways in macrophages during early infection [63]. The importance of apoptosis during PRRSV infection is controversial. A predominance of transcripts leading to prolonged cell survival has been reported [64], and a similar trend has been reported by a separate group [65]. Moreover, the absence of apoptosis during PRRSV infection has been observed in MARC-145 cells [66] and HeLa cells [67].

The co-expression analysis corresponding to the pig specific response to PRRSV infection revealed eight clusters (Additional file 5: Table S5-B, Additional file 6: Figure S1). Mapping the probes within each cluster onto the IPA revealed some canonical pathways that were present in the pig specific response to PRRSV infection like Regulation of eIF4 and p70S6K Signaling and Glucocorticoid Receptor Signaling. However, other canonical pathways emerged in this co-expression analysis and were not among the top canonical pathways when all the genes were submitted to IPA without clustering analysis (data not shown).

The most significant transcription factor during the pig specific response to PRRSV was HMGB1 (Figure 2B) that to our knowledge was not previously identified in the pig immune response to PRRSV infection. HMGB1 is necessary for the host innate recognition of viruses nucleic acids [68]. Moreover, this transcription factor is a crucial regulator of the fate and function of dendritic cells (DCs) [69] that connect innate and acquired immune responses. This feature could be found in our data also via the enriched biological function, “cell to cell signalling and interactions” (Additional file 4: Table S4B) and which involve CCL2, CCL3, CCL4, CD83, CD86, HMGB1, IL1A, IL8, MRC1, TLR2 and TLR4. HMGB1 might be a possible target of the PRRSV to manipulate the host immune response and eventually to generate an immuno-suppression.

Interferon regulatory factors, IRF1, IRF3, IRF5 and IRF8 were all involved in pig specific response to PRRSV infection (Figure 2C). IRF family proteins control expression of IFN-α and IFN-β-regulated genes that are induced by viral infection [70,71]. In addition, NFkB, EGR1, BCL3 and PYCARD were among the top transcription factors involved in pig immune response to PRRSV infection. NFkB was up-regulated during pulmonary infection by PRRSV [72]. SREBF1 and PYCAR are involved in response to stress [73,74] as well as in many viral infections via lipid metabolism regulation [75,76].

## Conclusions

To the best of our knowledge, this is the first time that very disparate microarray data corresponding to pig immune response challenges were combined statistically to identify common and specific genes and pathways between the general and specific immune response to PRRSV.

The regulation of translation and cell death were found to be the principal features of the pig global immune response to diverse challenges. In addition, most of the biological functions highlighted during this analysis corresponded to multiple aspects of translation, response to stress, as well as cell death. Specifically, during this meta-analysis we highlighted a significant alteration of the host cell homeostasis, which enhances the endoplasmic reticulum (ER) to send signals of stress. This might occur among other transcription factors via XBP-1 signalling. For the first time, we identified some transcription factors (MYCN, MYC and NFE2L2) that have been revealed to be highly significant in this analysis and their effect could be investigated in more detail.

For the pig specific response to PRRSV infection, we reported the involvement of well established immune responses like TREM1, toll-like signalling as well as the activation of the communication between innate and adaptive immune response and the regulation of anti-inflammatory response via glucocorticoid signalling. The implication of TREM1 during the PRRSV infection warrants specific attention, as other RNA viruses have been reported to act via this pathway. The potential involvement of the HMGB1 transcription factor in the innate recognition of PRRSV nucleic acids has been reported herein and potential implications discussed. Furthermore, interferon regulatory factors, IRF1, IRF3, IRF5 and IRF8 were all involved in pig specific response to PRRSV infection.

## Competing interests

The authors declare no competing financial interests.

## Authors’ contributions

BB wrote the paper, collected the microarray data, and performed the meta-analysis. CKT helped to conceive the meta-analysis project, supervised the probe annotation work, helped to write the paper. Z-LH performed the probes mapping into IPA and NCBI databases. JR helped to conceive the meta-analysis project as well as the microarray data to be included in the study. TAA helped to draft the manuscript and provided us with unpublished microarray data to be included in this study. AA helped in collecting and organising the microarray data. SB helped to conceive the meta-analysis work, coordinated the work, helped in writing the manuscript, analysis and interpretation of data. All authors read and approved the final manuscript.

## Supplementary Material

Additional file 1: Table S1 Summary of the microarray datasets on pig immune response included in the meta-analysis. Microarrays datasets included in meta-analysis of pig response to PRRSV infection are indicated in the column “Studies” by the word “PRRSV”. Data used for co-expression clustering are indicated in the column “Platform” by the symbol **. Click here for file

Additional file 2: Table S2 Probes used in global immune response : A. Probes used in global immune response and their corresponding p-values from Pointillist output. B. Probes significantly involved in global immune response mapped to Iowa Porcine Assembly. C. Probes significantly involved in global immune response mapped into Ingenuity pathways Analysis (IPA) . Click here for file

Additional file 5: Table S5 Clustering by co-expression analysis of the significant genes in the meta-analysis corresponding to the : A. Pig global immune response. Two clusters have been found, B. Pig specific response to PRRSV infection. Eight clusters have been found. Click here for file

Additional file 3: Table S3 Top five affected canonical pathways during pig global immune response and pig specific response to PRRSV infection. A. Top 5 affected canonical pathways and corresponding affected genes identified with IPA for the meta-analysis of the pig global immune response. B. Top five affected canonical pathways and corresponding affected genes identified with IPA for the meta-analysis of the pig PRRSV infection. Click here for file

Additional file 4: Table S4 Top five affected biological function “Molecular and cellular Functions” and corresponding affected genes for pig global immune response and pig specific response to PRRSV infection. A. Top five affected biological function for pig global immune response. B. Top five affected biological function for pig specific immune response to PRRSV infection. Click here for file

Additional file 6: Figure S1 (A) Heatmap representation of the gene expression network corresponding to the significant genes in pig global immune response (A.1) and pig specific response to PRRSV infection (A.2). This representation allows the visualization of modules related to the gene expression network, and how closely any two modules are related. (B) Hierarchical clustering of genes corresponding to the pig global immune response (B.1) and pig response to PRRSV infection (B.2) as well as visualization of gene module partitioning. The colored bars correspond to the module designation for the clusters of genes. Click here for file

Additional file 7: Table S6 List of 5 top affected canonical pathways and corresponding affected genes identified with IPA for the co-expression analysis of the pig global immune response. Click here for file

Additional file 8: Figure S2 MYC (A) and MYCN (B) transcription factors and their target genes found in the gene list corresponding to pig global immune response. The two transcription factors estimation was done using the IPA “transcription factor estimation” feature. Note that that the MYCN and MYC transcription factors explain three of the most significant canonical pathways reported in pig global immune response and shown in orange color (EIF2 Signaling, Regulation of eIF4, p70S6K Signaling, and mTOR Signaling). Click here for file

Additional file 9: Table S7 Significant probes in pig response to PRRSV infection. A. Significant probes in pig response to PRRSV infection and their corresponding p-values from Pointillist output, B. Probes significantly involved in pig response to PRRSV infection and their mapping into Iowa Porcine Assembly, C. Genes significantly involved in pig response to PRRSV infection and their mapping into Ingenuity pathways Analysis (IPA). Click here for file

Additional file 10: Table S8 Significant genes repartition between the pig global immune response and the pig response to PRRSV infection: A. pig response to PRRSV infection, B. global immune response, C. Genes common between global response and response to PRRSV to infection. Click here for file
