# Supplementary material for: Pig immune response to general stimulus and to porcine reproductive and respiratory syndrome virus infection: a meta-analysis approach
Source: BMC Genomics. 2013 Apr 3;14:220. doi: 10.1186/1471-2164-14-220 (PMC3623894; doi:10.1186/1471-2164-14-220)
Supplement: Additional file 1: Table S1 — Summary of the microarray datasets on pig immune response included in the meta-analysis. Microarrays datasets included in meta-analysis of pig response to PRRSV infection are indicated in the column “Studies” by the word “PRRSV”. Data used for co-expression clustering are indicated in the column “Platform” by the symbol **. [file 1471-2164-14-220-S1.doc]

| **Studies** | **Access_number** | **Description** | **Number of arrays** | **Number of eliminated arrays after quality check** | **Platform** | **Time** | **Breed** | **References** |
| --- | --- | --- | --- | --- | --- | --- | --- | --- |
|  | GSE11787 | Porcine spleen_Haemophilus Parasuis infected_7days | 6 | 0 | Affymetrix Porcine Genome Array** | Spleen: T=7 days post infection (P.I) | Commercial Herds | [1] |
| PRRSV | GSE12194 | Differential expression in lung and lymph node of pigs with high and low responses to infection with PRRSV | 112 | 0 | Qiagen-NRSP-8 porcine oligo array | Lung and Lymph node: T=14 days P.I | Idex and Line (Petry et al 2005) | [2] |
|  | GSE13134 | Transcriptional profiling at different sites in lungs of pigs during acute bacterial respiratory infection | 35 | 2 | DJF Pig 55K v1 | Lung: T=14-18 hours P.I | Danish Landrace/Yorkshire/Duroc cross | [3] |
|  | GSE13515 | Amniotic fluid and necrotizing enterocolitis in preterm pigs | 17 | 0 | Porcine oligonucleotide microarray version 4 (POM4) (Condensed version) | NA | NA | NA |
|  | GSE13516 | Temporal development of necrotizing enterocolitis in the preterm pig | 26 | 0 | Porcine oligonucleotide microarray version 4 (POM4) (Condensed version) | T=0 (no feed); T=8-24 hours (colostrum feed); T=8-24 hours (formula feed) | NA | NA |
|  | GSE14373 | Ischemia reperfusion injury (IRI) in organ transplantation and the effects of carbon monoxide treatment | 18 | 2 | Affymetrix Porcine Genome Array** | T=0 (naive); T=4 hours-untreated; T=4 hours treated; T=24 hours untreated; T=24hours treated | NA | NA |
|  | GSE14758 | Expression data from mediastinal lymph nodes of piglets experimentally infected with porcine circovirus type 2 (PCV2) | 24 | 2 | Affymetrix Porcine Genome Array** | MLN: T=1-2-5-8-29 hours P.I | Landrace CDCD | [4] |
|  | GSE14790 | Expression data from blood samples of piglets experimentally infected with porcine circovirus type 2 (PCV2) | 39 | 0 | Affymetrix Porcine Genome Array** | LWB: T=0-7-14-21-29 hours P.I | Landrace CDCD | [4] |
|  | GSE15256 | The Impact of Environment on Microbial Diversity and Global Transcriptional Responses in the Developing Pig Gut | 54 | 2 | Affymetrix Porcine Genome Array** | T=5-28-56 days | Large White × Landrace pig | [5] |
|  | GSE15766 | Gene expression profiles of European wild boar naturally infected with Anaplasma phagocytophilum. | 5 | 1 | Affymetrix Porcine Genome Array** | NA | European wild boar naturally infected | NA |
|  | GSE17320 | Transcriptome analysis of porcine PMBCs after stimulation by LPS or PMA/ionomycin using SLA-RI/NRSP8-13K chip | 28 | 0 | SLA/Immune Response/NRSP8 Pig 70 mers Oligonucleotides 3.8K + 13.3K v1 | T=24 hours P.I | Large White | [6] |
|  | GSE17492 | Preliminary characterization of gene expression in European wild boar naturally infected with Brucella suis | 6 | 0 | Affymetrix Porcine Genome Array** | NA | European wild boar naturally infected with Brucella suis | NA |
|  | GSE19126 | Comparison of Gene Expression in Lung of Pigs Exhibiting Different Susceptibilities to Glasser's Disease | 48 | 1 | Operon Pig 14.4K genome microarray v1.0.2 | T=24 hours P.I.; 72 hours P.I | Fully Resistant and Fully susceptible animal | [7] |
|  | GSE21071 | Cystic Fibrosis Pigs Develop Lung Disease and Exhibit Defective Bacterial Eradication at Birth | 40 | 1 | Affymetrix Porcine Genome Array** | NA | NA | [8] |
|  | GSE22311 | Expression data from pig mesenteric lymph nodes | 2 | 0 | Affymetrix Porcine Genome Array** | T=8 weeks P.I | NA | [9] |
|  | GSE2339 | Detection of gene expression changes in S. Choleraesuis infected porcine lung using an oligonucleotide microarray | 9 | 0 | Qiagen-NRSP-8 porcine oligo array | T=0-24-48 P.I | NA | [10] |
|  | GSE24889 | Understanding Streptococcus suis serotype 2 infection in pigs through a transcriptional approach | 18 | 5 | Affymetrix Porcine Genome Array** | T=24 hours P.I | NA | NA |
| PRRSV | GSE25120 | Identifying putative candidate genes and pathways involved in immune responses to porcine reproductive and respiratory syndrome virus (PRRSV) infection | 16 | 1 | pigoligoarray (http://www.pigoligoarray.org/) | T=4 days: T=7days; T=14 days P.I | NE Idex Line (I); Hamphshire Duroc (HD) | [11] |
|  | GSE4577 | Early pig response to experimental infection with Actinobacillus pleuropneomuniae | 16 | 0 | DIAS_PIG_55K2_v1; DIAS_PIG_27K2_v2 | T= 24 hours P.I | NA | [12] |
|  | GSE7232 | Transciptome profiling of small intestinal epithelial crypt and villi in germfree versus conventional neonatal piglets | 32 | 0 | spotted oligonucleotide:Intestinal epithelial crypt and villi in conventional relative to germfree pig | T=14 days from birth | NA | [13] |
|  | GSE7313 | Expression data from non-infected and Salmonella Typhimurium infected mesenteric lymph nodes | 15 | 1 | Affymetrix Porcine Genome Array** | T=8-24-48 hours for acute infection; T=21 hours for chronic infection | NA | [14] |
|  | GSE9259 | PrV/PK15 kinetics_QiagenNRSP8 slides | 32 | 0 | Qiagen-NRSP-8 porcine oligo array | T=1-2-4-8 hours P.I. | NA | [15] |
| PRRSV | E-MEXP-1350 | Transcription profiling of pig alveolar macrophages infected with Porcine Respiratory Reproductive Syndrome Virus | 21 | 1 | Affymetrix Porcine Genome Array** | T=0-1-3-6-9-12 hours | NA | [16] |
|  | E-MEXP-1756 | Transcription profiling of porcine alveolar macrophages in response to Streptococcus suis serotype 2 | 18 | 2 | Affymetrix Porcine Genome Array** | T=0, 2, 5, 10, 15, 60 hours | NA | NA |
|  | E-MEXP-2376 | Transcription profiling of pig brain and lung to natural infection by Pseudorabies virus (used human array) | 20 | 0 | CMR HsRefSet | NA | NA | NA |
|  | E-MEXP-991 | Transcription profiling of porcine alveolar macrophages (PAM) after antibody-mediated crosslinking of sialoadhesin (Sn, Siglec-1) | 12 | 1 | Affymetrix Porcine Genome Array** | T=2 | NA | [16] |
| PRRSV | E-MTAB-505 | Transcription profiling by array of porcine alveolar macrophages in a time course after infection with porcine | 47 | 6 | Array design A-AFFY-75 - Affymetrix GeneChip Porcine Genome Array ** | T=0-2-4-8-12-16-24-30 hours P.I | Landrace, Pietrain | [17] |
| PRRSV | GSE26642 | Pathology and Protective Immune Response in Pigs Infected with Porcine Reproductive and Respiratory Syndrome Virus (PRRSV) of High and Low Virulence | 70 | 0 | Swine Protein-Annotated Oligonucleotide Microarray | NA | NA | NA |
| PRRSV | Ait Ali et al (not published) | In-vivo infection with PRRSV | 23 | 2 | Array design A-AFFY-75 - Affymetrix GeneChip Porcine Genome Array ** | T=2-6-13-27-48 days P.I | 2 breeds (BC and D) | NA |

**References corresponding to supplemental Table 1**.

[1]. Chen H, Li C, Fang M, Zhu M et al: Understanding Haemophilus parasuis infection in porcine spleen through a transcriptomics approach. BMC Genomics 2009, 5:10:64.

[2]. Bates JS, Petry DB, Eudy J, Bough L, Johnson RK: Differential expression in lung and bronchial lymph node of pigs with high and low responses to infection with porcine reproductive and respiratory syndrome virus. J Anim Sci 2008 , 86:3279-89.

[3]. Mortensen S, Skovgaard K, Hedegaard J, Bendixen C et al: Transcriptional profiling at different sites in lungs of pigs during acute bacterial respiratory infection. Innate Immun 2011, 17:41-53.

[4]. Tomás A, Fernandes LT, Sánchez A, Segalés J: Time course differential gene expression in response to porcine circovirus type 2 subclinical infection. Vet Res 2010, 41(1):12.

[5]. Mulder IE, Schmidt B, Stokes CR, Lewis M et al: Environmentally-acquired bacteria influence microbial diversity and natural innate immune responses at gut surfaces. BMC Biol 2009 , 20,7:79.

[6]. Gao Y, Flori L, Lecardonnel J, Esquerre D, Hu ZL, Tellaud A, Lemonnier G, Lefevre F, Oswald IP, Rogel-Gaillard C: Transcriptome analysis of porcine PBMCs after in vitro stimulation by LPS or PMA/ionomycin using an expression array targeting the pig immune response. BMC Genomics. 2009, 10:546.

[7]. Wilkinson JM, Sargent CA, Galina-Pantoja L, Tucker AW: Gene expression profiling in the lungs of pigs with different susceptibilities to Glässer's disease. BMC Genomics 2010, 29,11:455.

[8] Stoltz DA, Meyerholz DK, Pezzulo AA, Ramachandran S et al: Cystic fibrosis pigs develop lung disease and exhibit defective bacterial eradication at birth. Sci Transl Med 2010 ,28,2:29.

[9]. Tian F, Lin D, Wu J, Gao Y et al: Immune events associated with high level protection against Schistosoma japonicum infection in pigs immunized with UV-attenuated cercariae. PLoS One 2010 ,15, 5:e13408.

[10]. Zhao SH, Kuhar D, Lunney JK, Dawson H et al: Gene expression profiling in Salmonella Choleraesuis-infected porcine lung using a long oligonucleotide microarray. Mamm Genome 2006 ,17(7):777-89.

[11]. Wysocki M, Chen H, Steibel JP, Kuhar D, Petry D, Bates J, Johnson R, Ernst CW, Lunney JK: Identifying putative candidate genes and pathways involved in immune responses to porcine reproductive and respiratory syndrome virus (PRRSV) infection. Anim Genet. 2012 ,43(3):328-32.

[12]. Hedegaard J, Skovgaard K, Mortensen S, Sørensen P et al: Molecular characterisation of the early response in pigs to experimental infection with Actinobacillus pleuropneumoniae using cDNA microarrays. Acta Vet Scand 2007, 27,49:11.

[13]. Chowdhury S R , King D E , Willing B P , Band M R , Beever JE, Lane AB, Loor JJ, Marini JC, Rund LA, Schook LB, Van Kessel AG, and Gaskins HR: Transcriptome profiling of the small intestinal epithelium in germfree versus conventional pigs. BMC Genomics 2007, 27: 215.

[14]. Wang Y, Qu L, Uthe JJ, Bearson SM et al. Global transcriptional response of porcine mesenteric lymph nodes to Salmonella enterica serovar Typhimurium. Genomics 2007 ,90(1):72-84.

[15] Flori L, Rogel-Gaillard C, Mariani V, Lemonnier G, Cochet M, Hugot K, Chardon P, Robin S, lefevre F : A combined transcriptomic approach to analyse the dialog between Pseudorabies virus and porcine cells. Developments in Biologicals 2008, 132: 99-104.

[16] Genini S, Delputte PL, Malinverni R, Cecere M, Stella A, Nauwynck HJ, Giuffra E: Genome-wide transcriptional response of primary alveolar macrophages following infection with porcine reproductive and respiratory syndrome virus. J Gen Virol 2008, 89:2550-2564.

[17]. Ait-Ali T, Wilson AD, Carré W, Westcott DG, Frossard JP, Mellencamp MA, Mouzaki D, Matika O, Waddington D, Drew TW, Bishop SC, Archibald AL: Host inhibits replication of European porcine reproductive and respiratory syndrome virus in macrophages by altering differential regulation of type-I interferon transcriptional response. Immunogenetics 2011, 63(7):437-48.

** Experiment used in the analysis of co-expression (see Material and Methods)
