# Supplementary figures and images for: Pig immune response to general stimulus and to porcine reproductive and respiratory syndrome virus infection: a meta-analysis approach
Source: BMC Genomics. 2013 Apr 3;14:220. doi: 10.1186/1471-2164-14-220 (PMC3623894; doi:10.1186/1471-2164-14-220)

## Slide 1
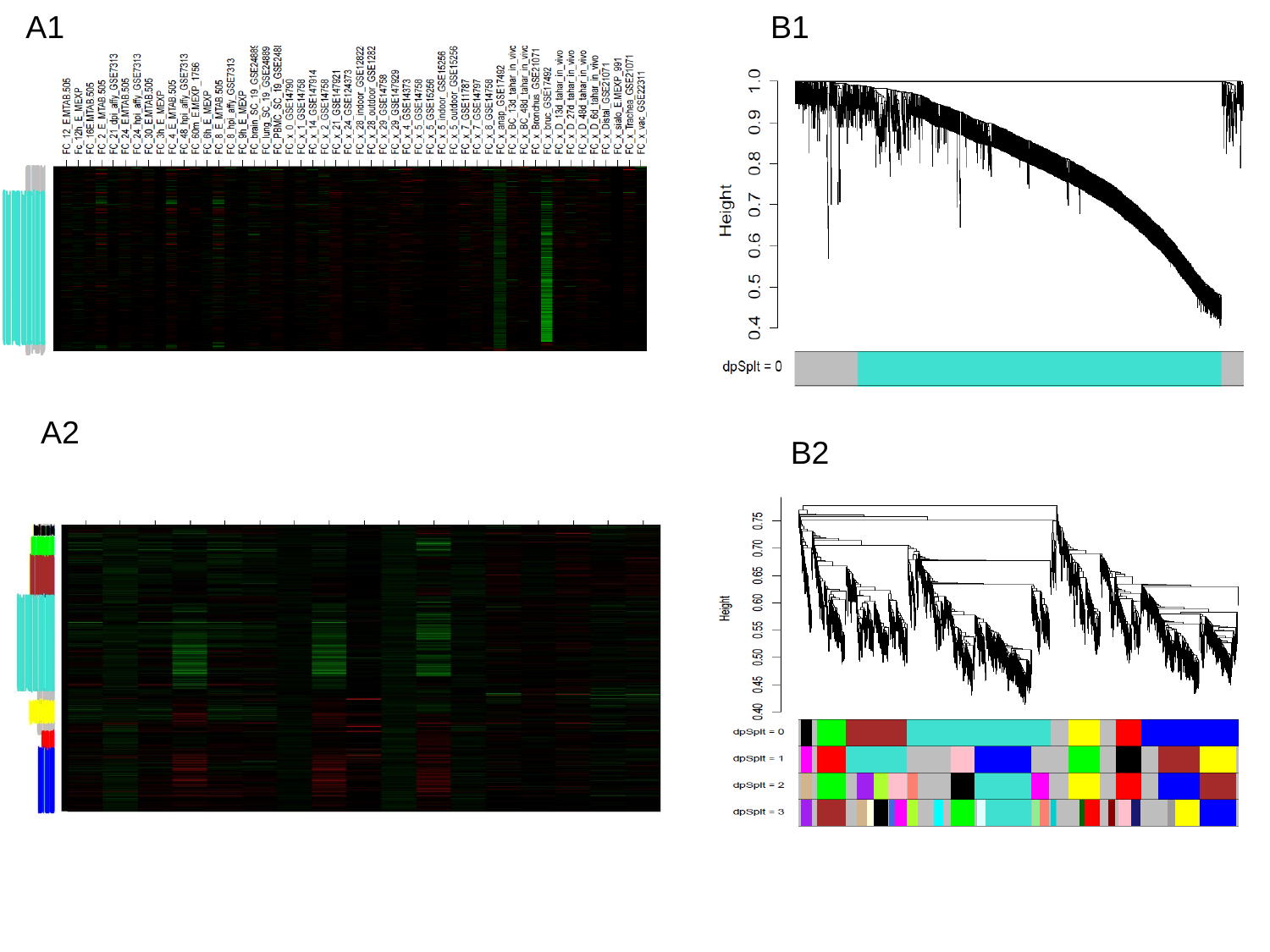

A1
B1
A2
B2

Supplement: Additional file 6: Figure S1 — (A) Heatmap representation of the gene expression network corresponding to the significant genes in pig global immune response (A.1) and pig specific response to PRRSV infection (A.2). This representation allows the visualization of modules related to the gene expression network, and how closely any two modules are related. (B) Hierarchical clustering of genes corresponding to the pig global immune response (B.1) and pig response to PRRSV infection (B.2) as well as visualization of gene module partitioning. The colored bars correspond to the module designation for the clusters of genes. [file 1471-2164-14-220-S6.ppt]

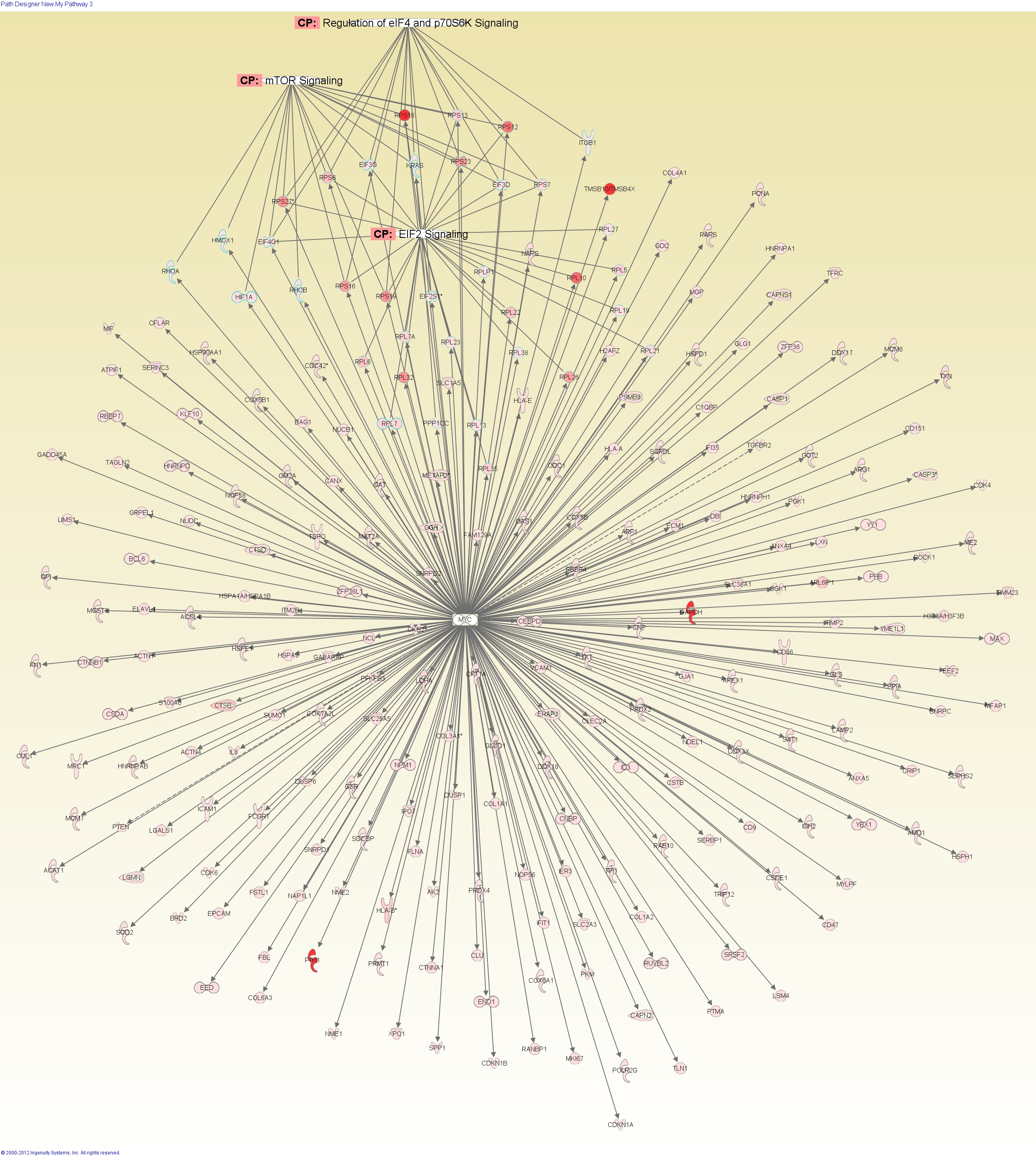


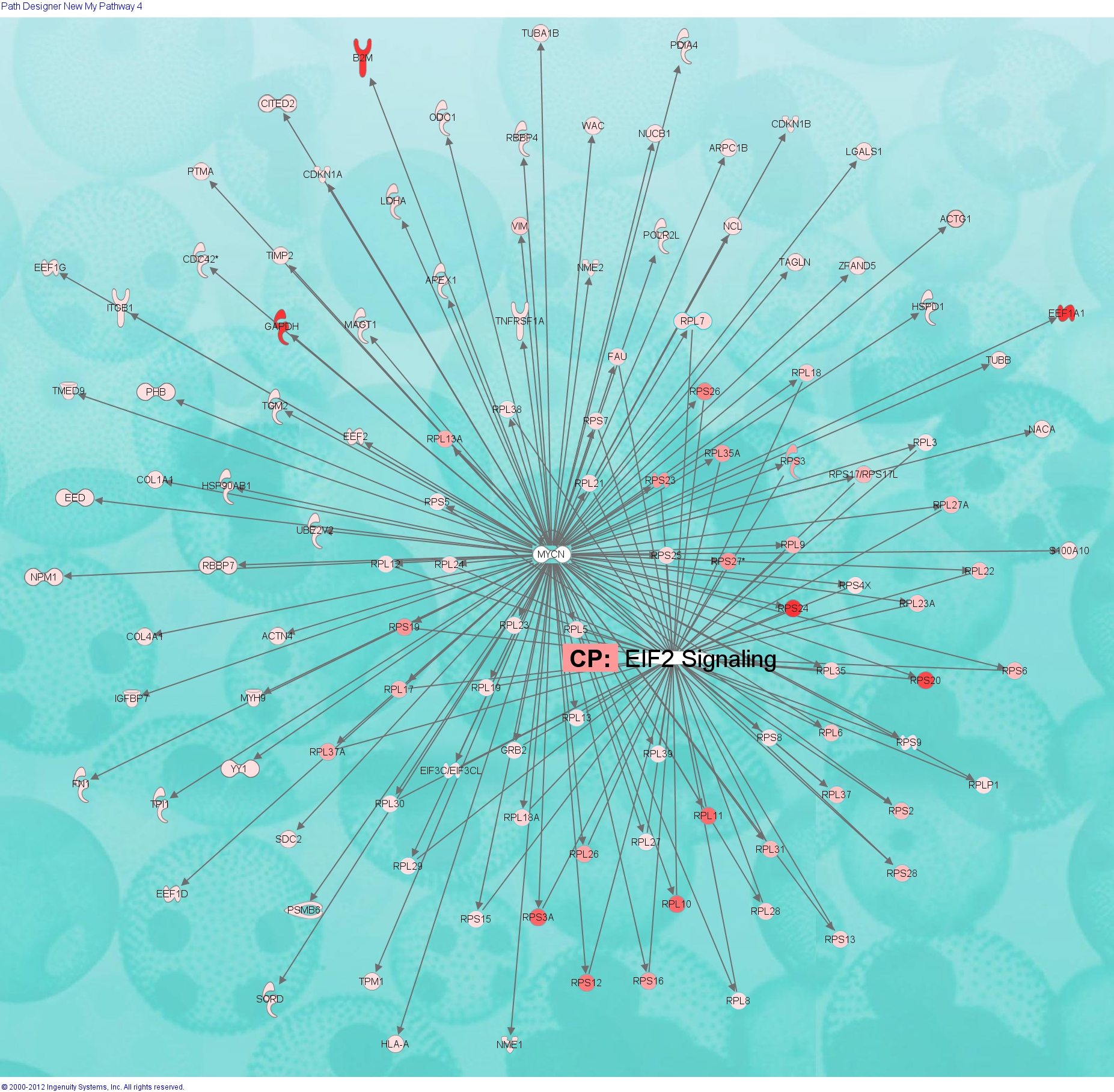

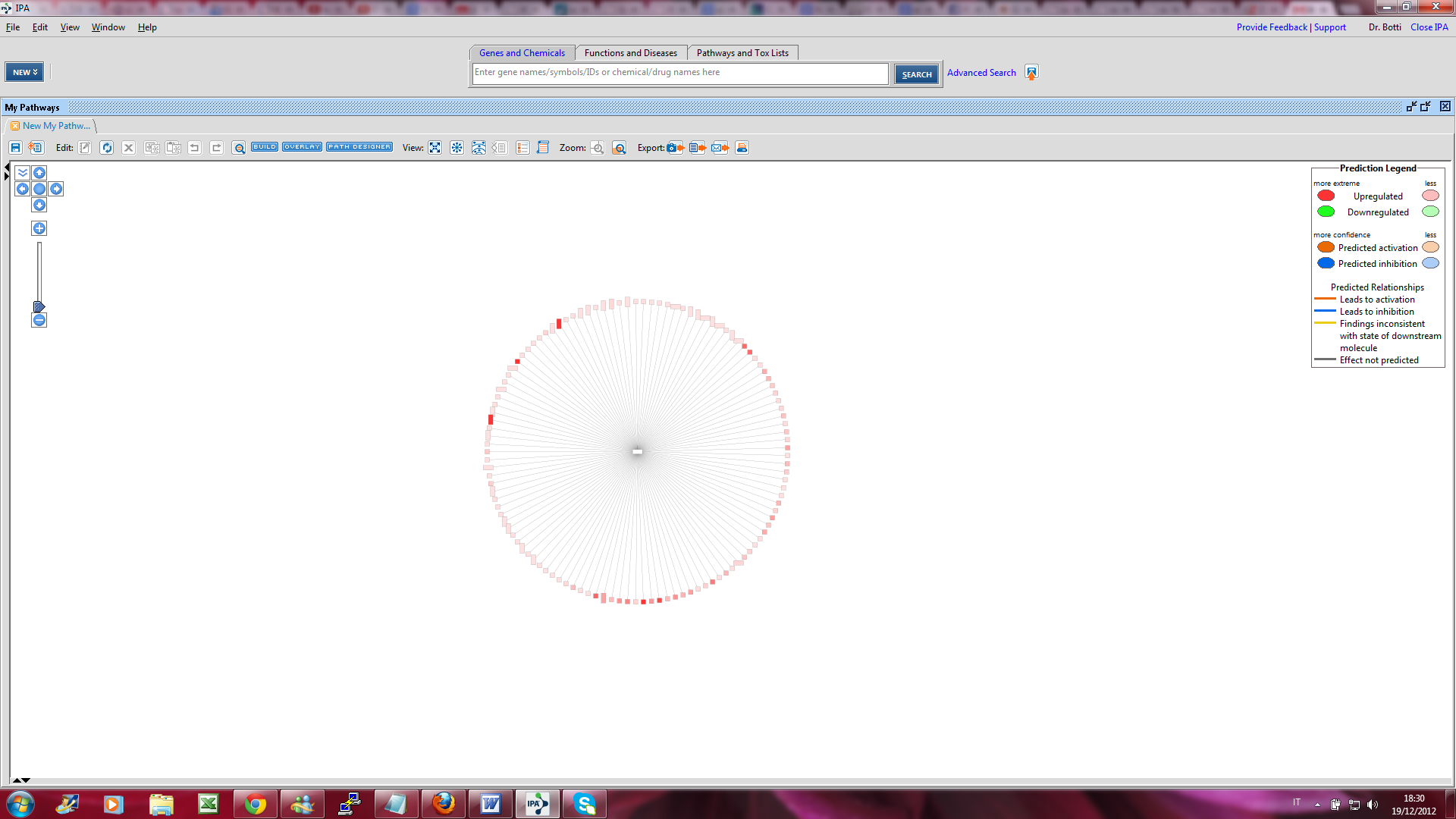

Supplement: Additional file 8: Figure S2 — MYC (A) and MYCN (B) transcription factors and their target genes found in the gene list corresponding to pig global immune response. The two transcription factors estimation was done using the IPA “transcription factor estimation” feature. Note that that the MYCN and MYC transcription factors explain three of the most significant canonical pathways reported in pig global immune response and shown in orange color (EIF2 Signaling, Regulation of eIF4, p70S6K Signaling, and mTOR Signaling). [file 1471-2164-14-220-S8.doc]
