# Supplementary material for: Pig immune response to general stimulus and to porcine reproductive and respiratory syndrome virus infection: a meta-analysis approach
Source: BMC Genomics. 2013 Apr 3;14:220. doi: 10.1186/1471-2164-14-220 (PMC3623894; doi:10.1186/1471-2164-14-220)
Supplement: Additional file 7: Table S6 — List of 5 top affected canonical pathways and corresponding affected genes identified with IPA for the co-expression analysis of the pig global immune response. [file 1471-2164-14-220-S7.doc]

| Top canonical pathways corresponding to the first clustering group (Tourquoise) | | |
| --- | --- | --- |
| Name | p-value | Ratio |
| EIF2 Signaling | 2.09E-78 | 91/200 |
| Regulation of eIF4 and p70S6K Signaling | 4.16E-33 | 50/174 |
| mTOR Signaling | 3.85E-27 | 50/210 |
| Mitochondrial Dysfunction | 4.2E-23 | 41/174 |
| Protein Ubiquitination Pathway | 2.01E-17 | 45/268 |
|  |  |  |
| Top canonical pathways corresponding to second clustering group (Grey) | | |
| Glycolysis I | 1.9E-05 | 6/43 |
| NRF2-mediated Oxidative Stress Response | 1.61E-04 | 10/192 |
| Ascorbate Recycling (Cytosolic) | 3.62E-04 | 3//13 |
| IL-12 Signaling and Production in Macrophages | 5.49E-04 | 8/156 |
| Clathrin-mediated Endocytosis Signaling | 9.99E-04 | 9/196 |
